# Thought leader perspectives on benefits and harms in precision medicine research

**DOI:** 10.1371/journal.pone.0207842

**Published:** 2018-11-26

**Authors:** Laura M. Beskow, Catherine M. Hammack, Kathleen M. Brelsford

**Affiliations:** Center for Biomedical Ethics and Society, Vanderbilt University Medical Center, Nashville, Tennessee, United States of America; University of Toronto, CANADA

## Abstract

Precision medicine research is underway to identify targeted approaches to improving health and preventing disease. However, such endeavors raise significant privacy and confidentiality concerns. The objective of this study was to elucidate the potential benefits and harms associated with precision medicine research through in-depth interviews with a diverse group of thought leaders, including primarily U.S.-based experts and scholars in the areas of ethics, genome research, health law, historically-disadvantaged populations, informatics, and participant-centric perspectives, as well as government officials and human subjects protections leaders. The results suggest the prospect of an array of individual and societal benefits, as well as physical, dignitary, group, economic, psychological, and legal harms. Relative to the way risks and harms are commonly described in consent forms for precision medicine research, the thought leaders we interviewed arguably emphasized a somewhat different set of issues. The return of individual research results, harm to socially-identifiable groups, the value-dependent nature of many benefits and harms, and the risks to the research enterprise itself emerged as important cross-cutting themes. Our findings highlight specific challenges that warrant concentrated care during the design, conduct, dissemination, and translation of precision medicine research and in the development of consent materials and processes.

## Introduction

Research is underway in the U.S. and around the world to accelerate progress on precision medicine, including the use of genomic, environmental, and lifestyle information to identify new and more targeted ways to improve the diagnosis, treatment, and prevention of disease [1, 2]. Initiatives such as the *All of Us* Research Program [3] offer opportunities for discovery and innovation on an unprecedented scale. However, such research platforms also raise significant privacy and confidentiality concerns, particularly as rapidly-evolving scientific and technologic advances have made it impossible to guarantee de-identification of genomic and other data [4, 5]. These issues require careful resolution not only for the protection of research participants, but for the sake of trust in the research enterprise itself [6].

To this end, we conducted a program of empirical research on the scope of confidentiality risks and protections applicable to large-scale gene-environment interaction research, as well as how these are and should be described to prospective participants. This included in-depth interviews with a diverse group of thought-leaders—prominent individuals based primarily in the U.S. uniquely positioned to identify both a depth and breadth of critical issues at the forefront of this swiftly changing landscape. Here we report their perspectives on the potential benefits and harms associated with precision medicine research.

## Materials and methods

### Participants

We conducted semi-structured interviews in the U.S. with nationally recognized thought leaders from an array of stakeholder groups likely to have diverse perspectives and experiences with respect to confidentiality and genome research, including:

- ELSI research (ELSI): Scholars who study ethical, legal, and social issues in genome science
- Ethics (Ethics): *e*.*g*., directors of centers for bioethics
- Federal government (Government): Individuals in relevant positions in the federal government
- Genome research (Research): Bench science and medical genomics researchers
- Health law (Law): *e*.*g*., directors of centers for health law
- Historically-disadvantaged populations (Historically-Disadvantaged): Scholars who study issues related to Historically-Disadvantaged populations
- Human subjects protections (Human Subjects): *e*.*g*., leaders of national organizations related to human subjects protections
- Informatics (Informatics): Bioinformatics, clinical and medical informatics experts
- Participant-centric approaches (Participant-Centric): Leaders in participant-centric approaches to research

We used stratified purposive sampling to interview at least six thought leaders per group, the minimum expected to reach saturation [7]. Through team discussion and personal networks, we identified prospective participants based on leadership positions in prominent organizations, institutions, and studies across the U.S., as well as authorship of highly influential papers on relevant topics. We expanded our sample by referral sampling [8], *i*.*e*., asking participants to suggest others whom we might consider for an interview.

### Instrument development

We developed a semi-structured interview guide centered around privacy and confidentiality issues and solutions in a hypothetical big data study—called the “Million American Study” (**Box 1**)—that involved extensive characterization (including whole genome sequencing) of biospecimens, ongoing collection of information from electronic health records, and real time monitoring of lifestyle and behavioral information through mobile devices. Interview topics included benefits, risks, and harms; informed consent, including emerging models of dynamic and open consent; and the strengths and limitations of a range of general and specific approaches to protecting confidentiality. The final instrument (available upon request), after refinements based on pilot testing, consisted of 19 questions; here we report findings in response to these two questions:

Imagine that your family members and close friends are all at a gathering together. The conversation turns to the “Million American Study” that has been in the news recently. Everyone is eager to hear your thoughts about whether they should consider signing up to be in this study.

…

3. How would you describe to your family and friends the benefits of participating in something like this?
4. How would you describe to your family and friends the primary risks of participating in the Million American Study?

*Probe*: You mentioned primarily risks to individuals; are there any risks to families and/or communities that you would tell your family and friends about?

**Box 1. The Million American Study (a hypothetical scenario)**

The Million American Study (MAS) is a large-scale research endeavor to improve understanding of health and to find new ways to predict, detect, diagnose, treat, and prevent disease. Specifically, the aim is to compile comprehensive information from a cohort of one million Americans in a repository that will serve as a rich research resource for a wide variety of studies for decades to come.

MAS will seek to enroll a representative sample of U.S. adults reflecting diversity in terms of race and ethnicity, age, and sex. Those who agree to participate will give consent for:

- Extensive characterization (including whole genome sequencing) of biospecimens, such as blood
- Ongoing access to clinical data (such as medications, test results, and imaging) from electronic health records
- Real-time monitoring of lifestyle and behavioral information, such as physical activity and environmental exposures, through mobile health devices

At the time of consent, participants will be offered choices about whether they are willing to be re-contacted for various purposes, for example to provide additional information or specimens, or to receive individual research results. Participants will be able to withdraw consent for future use of their specimens and data, with the exception that data generated in past studies cannot be withdrawn, nor can specimens and data be withdrawn from studies already begun.

Specimens and data will be stored in coded form in a federal repository. A robust data security framework will be in place, including administrative, technical, and physical safeguards. There will be a centralized governance process, comprising participant representatives, researchers, health care providers, government officials, and other stakeholders to ensure overall accountability and responsible project management.

Multiple tiers of access to MAS data—from open to controlled—based on data type, data use, and user qualifications will be employed. For example, certain information, such as some aggregate results, will be publicly available. Access to other information will be available to qualified researchers from academic, non-profit, and for-profit entities, in the U.S. and around the world, through application to a Data Access Committee. For approved projects, Material Transfer Agreements will be used to ensure that data and specimens are used and shared for authorized purposes only, and that privacy and security safeguards are maintained.

Information will be publicly available concerning how MAS cohort data and specimens are being used, including information about ongoing studies and summaries of research findings.

Adapted from Collins FS, Varmus H. New Engl J Med 2015; Khoury MJ, Evans JP. JAMA 2015

The Duke University Health System and the Vanderbilt University Medical Center Institutional Review Boards deemed this research exempt under 45 CFR 46.101(b)(2).

### Data collection

We emailed prospective interviewees an invitation to participate, including a study information sheet. Among those who indicated willingness, we provided a description of the “Million American Study” and an outline of the interview topics in advance.

The interviews were conducted by telephone between September 2015 and July 2016 by three members of the research team. At the beginning of each interview, we reviewed the study information sheet and obtained the participant’s verbal agreement to participate. Interviews ranged in length from 30 to 120 minutes, with an average length of approximately one hour. With participants’ permission, interviews were audio-recorded and professionally transcribed. Participants were offered $100 compensation for their time.

### Data analysis

We uploaded transcribed interviews into qualitative research software, NVivo 11, and used an overarching grounded theory approach and constant comparison to code and analyze the data [9, 10]. Specifically, two team members first created a structural codebook to delineate each question and corresponding response based on the interview guide. They then developed an initial codebook of thematic or content codes, based on substantive content identified in independent reviews of four transcripts. They independently applied these codes to a fifth transcript and then compared the results to modify codes and code definitions as needed. They followed this iterative process with additional transcripts until they achieved at least 80% inter-coder agreement in structural and content code application. The remaining transcripts were then divided between the two coders; each independently coded every sixth interview to ensure inter-coder agreement remained at a minimum of 80%.

Once all data were coded, the team systematically generated narrative summaries of relevant structural and content codes to explore the range of thematic responses and to identify additional sub-themes [11, 12]. Each summary was reviewed by at least one other team member who read the corresponding NVivo code reports to identify and confirm agreement in sub-theme identification and the synthesis itself. See **S1 Appendix** for additional methods-related details.

### Risks versus harms

Our interview Question 4 was stated to refer colloquially to “risks.” In the analytic process, however, we endeavored to identify content reflective of more formal definitions of risk versus harm [13]. With regard to *risk*, we looked for elements of interviewees’ responses that concerned the probability or likelihood of an event occurring, as well as specific sources of risk. With regard to *harm*, we looked for responses describing the actual adverse events that could result. Because of the density and complexity of thought leaders’ input on these issues, we separately published our findings concerning risk associated with participation in the Million American Study [6], which comprised four broad categories: (1) unintended access to identifying information; (2) permitted but potentially unwanted use of information; (3) risks based on the nature of genetic information; and (4) risks arising from the longitudinal study design. Here we focus on findings concerning benefits and harms.

## Results

We interviewed 60 primarily U.S.-based thought leaders, representing a wide array of perspectives and demographic diversity (**Table 1**).

**Table 1:** Participant characteristics (n = 60).

	*n*	(%)
Perspective:		
ELSI research	6	(10.0)
Ethics	7	(11.7)
Federal government	7	(11.7)
Genome research	7	(11.7)
Health law	6	(10.0)
Historically-disadvantaged populations	7	(11.7)
Human subjects protections	7	(11.7)
Informatics	6	(10.0)
Participant-centric approaches	7	(11.7)
Academic Degrees:		
MPH / MSPH	7	(11.7)
Other master’s degree (*e*.*g*., MA, MS, MBA)	23	(38.3)
JD, LLB / LLM	18	(30.0)
PhD	35	(58.3)
MD	16	(26.7)
RN	2	(3.3)
Based in:		
United States	58	(96.7)
Other (Canada, UK)	2	(3.3)
Gender (self-reported):		
Female	31	(51.7)
Male	29	(48.3)
Race (self-reported):		
American Indian or Alaska Native	2	(3.3)
Asian	5	(8.3)
Black or African American	3	(5.0)
Native Hawaiian or Other Pacific Islander	1	(1.7)
White	49	(81.7)
Ethnicity (self-reported):		
Hispanic or Latino	2	(3.3)

Interviewees described individual and societal benefits associated with our hypothetical Million American Study, as well as a variety of harms that could be classified as physical, dignitary or stigma, economic, psychological, or legal.

### Benefits

Nearly all thought leaders discussed potential benefits that could come from the Million American Study, including a range of health and non-health-related benefits to individual participants, as well as benefits to society from advancing scientific knowledge and improving human health.

### Individual benefits

For participants in our hypothetical study, most thought leaders predicted that *“the benefits are unlikely to be significant at the individual level”* (01, Human Subjects) and that although participation *“may help somebody in the future*, *it’s unlikely to help you”* (12, Law):

One should always emphasize in research that you probably won’t benefit. The reason to do this study is not because you will probably personally benefit. Most people don’t benefit from research individually, but as a society we have to pursue research or we’re never going to make progress and improve health and improve our systems. (44, Research)

A few mentioned the possibility of health benefits ancillary to study participation, such as *“real-time monitoring of lifestyle and other behavioral information*, *so that people can* [realize], *‘Oh my god*, *I only walked 16 steps today*, *I really need to walk more’”* (40, Human Subjects).

To the extent interviewees anticipated any direct health benefits, these were primarily expected to arise in the context of returning clinically actionable research results. Thought leaders typically acknowledged that such results (and the associated health benefits) would likely accrue to only a small proportion of research participants:

There is a chance that individuals in that study could get results that could be stupendously beneficial—perhaps even life-saving—from being sequenced or other biomarkers. That could be extraordinarily beneficial… It may not happen to everyone, probably not even to most people. But it could happen, that’s a large potential benefit. (20, Research)

Depending on how the study is set up, if there is the opportunity for return of results, there may be some individual benefit. But even under the best estimates that we have at the present time, only about two to four percent of individuals will have a finding that we would currently consider to be actionable. (58, Research)

Even so, a few recognized the converse: that many people could theoretically benefit from learning that they do not have a deleterious gene variant:

It’s kind of nice to know that none of the major risk factors that we know about in the known high pathology genes, like Huntington’s and BRCA1 and 2 … that I don’t have any of those mutations that are strongly associated with disease outcomes. (52, ELSI)

Beyond the relatively high threshold of returning only those results that could lead to clinical action, some interviewees pointed to possible health benefits from receipt of research results indicating the need for lifestyle changes; for example, *“if someone finds out that their information suggests that they may be at risk of heart disease*, *they may want to think about exercising more and/or eating better”* (40, Human Subjects). Others were less certain that returning such results would lead to dramatic changes, particularly if accompanied by already well-known advice:

There’s always a potential that some individual result of yours is beneficial to you. Today, in general, that tends not to be the case. Most people who have whole-genome sequencing or genotyping through 23andMe don’t get information that radically changes their lives in any particular way… By and large, the things that you should do to help maximize your chances of avoiding bad things are the things that we all already know we ought to be doing: exercise more, eat less, eat better, don’t smoke, don’t drink too much, etcetera. (54, Ethics)

Deriving health benefits from any individual research results would depend on accurate interpretation of the data, which at least some thought leaders assumed would be provided by the study:

There is the potential for very direct personal gain by people having access to the data within their genomes and being part of a study that’s actively trying to figure out what all that information means, and the potential for them to have results directly interpreted and relayed back to them. (56, Research)

A few, however, remarked that both the interpretation and value of the results might differ for each participant, based on clinical context or other personal characteristics:

Many of the genomic findings that we used to think to be iron clad we now know to have very different meaning… For example, if you have the symptoms of hemochromatosis and you have a variant in HFE, a gene that’s linked to hemochromatosis, there’s an 80 percent chance that that variant is actually contributing to your hemochromatosis. If, on the other hand, you are otherwise healthy, and you get your HFE gene sequence … and there’s a variant in that gene, the chance that this is actually contributing to hemochromatosis, down the line, is less than one percent. And that’s been shown for multiple genes. (33, Informatics)

If you’re 80 years old, almost anything that could come out of that sequence in some ways has to be couched in the fact: ‘Well, okay, great; you’ve got an actionable gene. But you’re 80 years old.’ (58, Research)

Others noted that there is still a considerable amount to learn before much genomic data can be accurately interpreted…

There’s going to be so many variants of unknown significance. And for the most part, so little comfort, because it’s rare that it’s going to come back … ‘Hey, we did a whole genome sequence, and we figured you out!’ (23, Participant-Centric)

…and thus the benefit of receiving individual research results might lie in the future:

Right now we just don’t know what a lot of genetic variants are. We don’t really know what it means that people differ in these particular genetic ways. In the future, we may know more about that. And presumably if you get your data back, you’ll have it when, in the future, we may learn more about it. (54, Ethics)

Interviewees also discussed less tangible or non-health benefits for individual participants, such as the opportunity for altruism. As one observed, “*The indirect benefit is that altruistic one*, *to feel like you’re doing something that could help build knowledge that could help other patients”* (20, Research). Some thought leaders specifically framed this type of benefit around families and future generations, pointing out that having a personal connection to the goals of the research might be an important consideration for some people:

How people make decisions is by trying to strike some reasonable balance of benefits and harms, so the individual’s personal situation—they may have a bunch of relatives who died of cancer and nobody’s figured it out. That kind of person might say ‘risks be damned, I’m willing to try anything to figure out what’s going on in my family.’ (20, Research)

I think it’s very important to understand that the likelihood of someone individually benefiting quickly is small. So people need to feel comfortable thinking about this for the future or for their children or their grandchildren or for others. (50, Human Subjects)

Other interviewees, however, observed that the broad, open-ended nature of the study may make it difficult for prospective participants to form this connection and thus affect how they perceive the potential benefits:

The benefits are … very much based on altruism, and that ‘this will serve as a rich resource for a variety of studies for decades to come’ [paraphrasing Million American Study description]. All of that is a bit nebulous—the fact that they’re not actually narrowing it down, that it’s going to be used for all sorts of diseases… The benefits are largely uncertain at this point in time. (09, Participant-Centric)

It’s one of the inherent attributes of this kind of genomic research—really what you’re asking people to do is to sign up for an open-ended study where you don’t actually know what you’re going to do downstream. You don’t know what the benefits are going to be and you don’t what the risks are going to be. (20, Research)

Finally, a few thought leaders identified the individual benefit of deriving enjoyment from participation in the Million American Study:

It’s sort of interesting. It’s educational. It might be fun. You might get the warm fuzzies from feeling like you’re doing your part. (54, Ethics)

One interviewee’s response integrated many of these themes surrounding health and non-health benefits, particularly as they relate to individual research results, and highlighted the importance of setting realistic expectations concerning the prospect of direct benefit:

If my family asked me, ‘Are there personal benefits?’ … I would say ‘I would not anticipate that you’re going to get any personal benefit.’ And I can anticipate, thinking about some members of my family, that they might say, ‘Wait a second. They’re telling me that I’m going to be able to get access to all my data. Isn’t that cool?’ My answer to that would be, ‘If you like that kind of stuff, sure, you might derive some personal entertainment out of having access to all your data—but don’t pretend, though, don’t imagine that you’re going to get a health benefit out of this.’ I think the benefit to individuals participating, putting voluminous amounts of data in, as best we can tell, is going to be [that] they have access to their information and that might satisfy curiosity—but I don’t see people getting benefits beyond that. And the curiosity comes at a price. There’s the potential that they will get lots of information that they feel compelled to apply some meaning to in a way that may or may not be justified. In other words, the risk is that people will get information that they think is meaningful, health risk information, when in fact we don’t know if it is or not. (22, ELSI)

### Societal benefits

Thought leaders commonly described the major benefits of our hypothetical study as those occurring at a macro level, for *“the greater good*, *so to speak—the societal benefit that comes from combining their individual results with a million others*, *and learning what we can from that level”* (26, Human Subjects). Given the size and scope of the Million American Study, several interviewees even likened participation to a civic duty:

They need lots and lots of people to sign up, so they can actually do the kind of research to develop better understanding and better tools… You know, it’s like voting. Your individual vote may not matter, but if nobody votes, nothing works. (02, Government)

Part of the Million American idea is a little bit about good citizenship… It’s a little different than typical biomedical research. The Million American Study is more about being patriotic, invoking the idea that this is good for us as a group of people who live in the same society. (11, ELSI)

Thought leaders broadly defined the societal benefits as advancing scientific knowledge, with some believing the study *“has great potential to inform how diseases not only manifest themselves*, *but what the causes may be”* (55, Government). Many believed that the scale and duration of the Million American Study would help accomplish these goals, noting that *“one of the biggest roadblocks for research to date has been the lack of coordinated collection of a variety of data types across large populations of individuals”* (15, Ethics):

My initial reaction [to the study] is around the scientific possibilities and how much information could be put together or gleaned from looking at that number of individuals and studying them over the course of many years to observe what happens with regard to their health, and to try and identify biomarkers and/or genetic contributors to health and to disease. (21, Government)

A few, however, were uncertain that the sample size would be large enough…

I’m so jaded in terms of ‘Is it doable?’—but if I were to take a step into un-reality and say they actually *are* going to get meaningful phenotypic data, environmental data, and genotype data, there would be the opportunity of actually better understanding genetic contribution to disease… Certainly there’s huge value in the big data and I think the federal government is the only entity that can pull together big data to this degree. But at the same time, I think a million is too small. (34, Human Subjects)

…or had other doubts about assembling the cohort:

I think it has the potential to teach us a lot about the range of human genotypic and phenotypic information and could be quite useful for lots of interesting research projects. I think it’s going to be interestingly challenging to figure out who to include. You know, how do you get a representative sample of U.S. adults? It’s not so straightforward. (27, Government)

Assuming that the study could produce scientific advances, many thought leaders anticipated these would lead to better health and health care, to the *“opportunity to finally crack open the things that we need to crack open in order to start to get the right solutions in health”* (35, Participant-Centric). Some interviewees, however, were concerned that these benefits have been over-sold:

It has the potential for researchers to learn things that are valuable about people, health and otherwise. And that, in turn, ought to eventually trickle down and be something that improves everybody’s healthcare. Just a note of caution that that’s really speculative. Precision medicine has been incredibly hyped. And so this isn’t going to be the kind of situation where a million people contribute to this and next year we have a cure for Alzheimer’s or cancer. That’s not how it’s going to work. (54, Ethics)

A few thought leaders highlighted the translation of scientific knowledge into improved health as a specific hurdle:

We may find associations. We may even find causal relationships. That doesn’t mean that it’s going to rapidly translate into new therapies, new cures, and so on. I think there’s some degree of hype surrounding these kinds of endeavors. (12, Law)

In particular, several interviewees commented on whether the research would lead to improved health for all segments of society. A few were hopeful:

What I think is important is that all segments of the population need to be represented. It’s really being included and being represented in the research so that findings are relevant and more generalizable across populations versus really reflecting only the participation of one group. (60, Historically-Disadvantaged)

Others, however, had misgivings about whether some subgroups would benefit. Small sample sizes, even with high participation rates by the group, were one cause for concern:

I understand fully the intentions [and] I think it’s something that we should consider and think about in a positive light. However, I think we should also enter into it with some hesitation. Part of that hesitation has to do with the idea of, statistically, what are the chances of having enough participants from the [Indigenous] community for the benefit to actually … be interpretable for [Indigenous] people? Because we are such a small population and there are other populations that are very small. My hesitancy is just representation and benefit. (36, Historically-Disadvantaged)

Failures to translate scientific findings into interventions that actually address the health problems that subgroups disproportionately experience were another concern:

There’s lot of literature out there that shows how [specific Indigenous] people have worse health and worse outcomes. At some level, continuing to publish on that … is probably not that helpful. What most communities really want now is ‘Okay, yeah, we know all that, but what can be done to help that?’ (30, Historically-Disadvantaged)

Finally, even when interventions are available, there was concern that lack of access could exacerbate health disparities and mistrust in research:

What’s risky is asking people from different racial and ethnic backgrounds, low-income backgrounds, to participate in the study when they will not have access financially to the benefits of the information gained from the research. Because then ten years from now, people will be upset that their families contributed to the research and … there’s still a large gap in terms of outcomes. (10, Historically-Disadvantaged)

Overall, thought leaders recognized that attaining the societal benefits of advancing scientific knowledge and improving human health would require substantial social and political commitment to the research:

I would [want to] make sure that there was a bipartisan support for this initiative both in a budgetary sense and also in a moral and political sense… It’s trouble that nobody wants to go to unless they’re assured that the study is actually going to be finished. (29, Human Subjects)

Interviewees also recognized that achieving societal benefits must be balanced with the risks that individual participants are asked to take on:

This research study has broader societal benefits, but there’s not as much of a clear personal benefit for individuals to participate. So they’re going to have to make that trade-off of, is it worth it for them to take risk to their privacy to participate in a study like this for the benefit of society? (15, Ethics)

Many seemed to view this trade-off favorably, based on a perception of our hypothetical study as low personal risk/high societal reward:

I think there’s a lot of public benefit that can come from studies like these and of this scale. With appropriate protections and controls put in place, I think the risk-benefit ratio is quite favorable, and I’m in favor of studies like this. (26, Human Subjects)

Others, although convinced of the societal benefit, were less certain about the implications for individual participants:

I think the vision itself of being able to amass the health and genomic data and consumer device data of a million people has tremendous potential to provide real value and help us accelerate the advancement of biomedical knowledge very quickly, which is a huge benefit to society. But it may not be that much of a benefit to the individual, particularly considering the risk that the approach seems to be imposing on the individual. (51, Informatics)

### Physical harms

Approximately one-fourth of thought leaders identified physical harms that could come from the Million American Study. A few mentioned negligible potential harm from having blood drawn (*“nothing more than what you face every time you have an annual checkup or donate blood”* (24, Government)), but more importantly, interviewees discussed physical harms stemming from unwarranted, questionable, or premature actions based on research results.

### Research results and individual action

Recognizing that participants in our hypothetical study would be offered choices about getting individual research results, many thought leaders anticipated that return of results could bring about several kinds of harm. One even opined that the overall risks of the study *“largely hinge on whether results are returned or not”* (01, Human Subjects). With regard to physical harms specifically, interviewees were concerned that participants might take unwarranted medical action based on their individual results: *“go get medical tests*, *spend time and money*, *incur additional risks from other medical procedures … that really you would have been better off not* [doing]*”* (16, Ethics) and *“start paying money for tests and surgeries they don’t need”* (10, Historically-Disadvantaged). This concern was commonly based on the prospect of inaccurate, misinterpreted, or uncertain information. With regard to inaccurate information, some thought leaders cited errors related to biospecimen handling:

This is the real concern, in my view, about return of individual research results: unless samples [and] data are handled under very strict clinical-like conditions, that providing individual research results back can be a risk—return or release of information that may not be correct. (59, Government)

Another mentioned medical errors more generally:

As an example, in BRCA1, if one really is careful, that can be done well and communicated and actually improve that person’s health, reduce their risk for that outcome, but we all know that things go wrong. Things go wrong all the time in medicine. We make mistakes, we misinterpret things, and the impact of that unlikely screw-up is significant. (44, Research)

Several interviewees singled out false positive interpretations, *“and all that comes with that”* (26, Human Subjects):

We will see lots and lots of false positive associations. This is a predictable outcome of … looking for associations in voluminous data. That doesn’t mean we shouldn’t do that ever. It just means that it’s a very complex research effort and, in the meantime, if you’re feeding information back to individuals, there’s a distinct risk that you’re going to feed back non-casual associations or false positive associations that people will think mean something. They will think there may be reason to take action when in fact it’s a premature thing to do. (22, ELSI)

These descriptions were often accompanied by reference to actual experience: *“People have had breasts removed for BRCA variants that have been proven to be benign*. *People have terminated pregnancies for variants that we know for a fact are benign”* (33, Informatics).

With regard to uncertain information—for example, if participants chose to get back data that included variants of uncertain clinical significance—thought leaders worried that people *“might start interpreting it themselves … and jumping to conclusions”* (48, Law), and that there could be *“a substantial risk of chasing down stuff*, *putting yourself through procedures and expense and heartache and that sort of thing for not a lot of gain”* (16, Ethics).

Beyond physical harm stemming from unwarranted medical actions, a few interviewees described harms associated with medicalization in general, *i*.*e*., from return of research results leading to *“a series of interventions*, *designed to reduce risks that have risks of their own”* (08, ELSI):

If the study was going to return results to you that one thought were medically important, that then entangles you in the medical system. It might lead to treatments and drugs and tests and, of course, anytime you start inflicting modern medical care on a person, there are risks involved in that. It could take people down a path that leads to issues—all the issues and all the risks that come with medical care. (44, Research)

Among thought leaders who mentioned physical harms arising from return of research results, most said that the likelihood of such harm would depend on several factors. First and foremost were decisions about which results would be offered:

It depends upon how the study is designed, what the investigators … plan to offer back to the participants. If there’s no plan, if the upshot was ‘there’s no way we’re going to give you back any data,’ then [harm] would be very unlikely. If the upshot was ‘we’re going to give you back whatever you want or we’re going to give you back everything, you can access to your raw files if you want them,’ then it’s very likely. (16, Ethics)

Thus, some interviewees emphasized offering only clinically actionable results as a way to minimize the chance of harm: *“If we stayed within that defined set that people can act on*, *that’s in their best interest to know about*, *then I think the chances of harm from that kind of thing is pretty small”* (26, Human Subjects).

More broadly, study design and planning for communicating results was another common dependency influencing the likelihood of harm:

The devil is in the details. If this study is really designed carefully—and that’s a big asterisk, that’s a big caveat—I could easily see this study being designed poorly with the risks being high. But I’m going to assume that very thoughtful people who actually understand medicine, not just genetics, but who understand medicine, are designing it. If that’s the case, then I would say that the likelihood of harm to an individual would be unlikely. (44, Research)

### Research results and health system action

A few thought leaders alluded to downstream physical harms that could occur at a health system level, if healthcare providers or institutions make premature decisions based on research results. Specifically, they foresaw such harm if providers *withhold* healthcare services, based either on knowledge of an individual’s research results…

If your healthcare provider decides the most efficient way to cut costs after [getting] this information is to cut out some services you need, then that’s a very serious consequence. (37, ELSI)

…or on published aggregate results that, for example, suggest something about a particular group:

When ethnic groups are discovered to have genes that are pharmacogenomically relevant, making a person from that ethnic group likely to respond poorly to a particular drug, then those people from that group may be seen as difficult to treat or they may be denied access to a drug just because they’re a member of the group… In reality, there could be some [patients] who would benefit from it, but doctors are avoiding the drug in that population. (10, Historically-Disadvantaged)

Conversely, a few foresaw the possibility of physical harm if providers *implement* interventions with misguided justification:

One of the things I’m really concerned about is attempts to use big medical data predictively, because I think we’re actually really bad at doing that. People can jump the gun and think, ‘Well, maybe we ought to do some interventions to prevent something from happening.’ … We are sequencing more and more people who are generally healthy people and we keep finding things in their genomes where, ‘Gosh, that variant has been listed in all the databases as something that is strongly associated with having a serious health problem.’ Except, ‘Oops!’ Now we’re finding it in all sorts of healthy people. So now we have to reconsider the meaning of having that genetic variant. (19, Ethics)

### Dignitary and group harms

More than three-fourths of thought leaders discussed the potential for dignitary or group harm arising from the Million American Study. Of these, approximately 60% raised these types of harms unprompted, whereas the other 40% commented in response to the one-time prompt in our interview guide, “You mentioned primarily risks to individuals; are there any risks to families and/or communities that you would tell your family and friends about?” (see Methods).

### Dignitary harm

Thought leaders described the possibility of dignitary harm resulting from uses of biospecimens and data that participants find objectionable or that *“people didn’t like* [and] *don’t really want to contribute to”* (27, Government). In contrast to unintended access to stored materials—due to breach or hacking, for example—dignitary harm was seen as *“not accidental”* (11, ELSI), but rather a potential outcome of authorized uses that participants did not anticipate:

There are people who, in the abstract, are supportive of research. But if you go through a long list of possible uses of the information, they may be a little less comfortable with some of those uses. So, it depends on what the research from this biobank produces. They may be unhappy with what comes out of it. (12, Law)

Interviewees discussed a variety of reasons why a participant might object to particular uses:

You may be someone with very strong religious beliefs and there’s a study being done that is on a subject matter that may not fit in or accord with your belief. You may be a person who doesn’t think that it’s appropriate that research is done on intelligence and race, for instance. You may be someone who has a whole range of views about things… There’s a whole series of reasons why people might have concerns about certain types of research. (09, Participant-Centric)

They primarily attributed the potential for participants to suffer dignitary harm to the Million American Study being *“a pretty open-ended research endeavor”* (31, ELSI), a long-term effort relying on broad consent for future unspecified research. Participants’ loss of control over their biospecimens and data was a common theme, which several interviewees explicitly distinguished from harms to informational privacy:

This is really an autonomy risk. It’s not a privacy risk… It came from their body, and [people] believe they ought to have some say over what happens to it. (12, Law)

Just as important as those privacy risks is the loss of control that you would have over the use that’s being made of your biospecimens and information. That’s not something that hurts you in the same way, but it seems like it’s something a lot of people would be concerned about: just not knowing or having a voice in what’s being done with information about you. (37, ELSI)

Interviewees expressed a wide range of opinions about the likelihood and severity of dignitary harms resulting from unanticipated uses. For example, one predicted that the chance of objectionable use was high *“because there is so much research going on*, *and I think many people have really no idea”* (45, Participant-Centric). Conversely, another expected that the likelihood was low because of the size and visibility of the Million American Study:

I would be remiss not to mention [objectionable use] to friends and family. But I would then say, ‘The vast majority of research that’s done is benign. It’s about cardiovascular conditions, whatever. It’s not, ‘let’s create bioweapons.’ And especially: large-scale studies like this that are very high-profile are even less likely to involve those kinds of questions.’ (54, Ethics)

This interviewee went on to question both the wisdom of attempting to proscribe particular areas of research, as well as the actual consequences of objectionable use:

Any research results can be misinterpreted or misused. And conversely, a lot of research can result in serendipitous results that have beneficial uses that even the researchers never predicted. So in general I am very skeptical about eschewing certain research questions or certain types of research out of fear that it would be misinterpreted or misused. I just think that that’s generally a bad way to do science… If [objectionable research was an issue], it’s just sort of a complicity; it’s symbolic: technically your data contributed to this. But it’s not like that necessarily means you’re going be harmed in any way by it. (54, Ethics)

Another thought leader similarly reflected on the intangible nature of dignitary harms:

It’s a kind of harm that relates to your information being used in ways that you didn’t agree, but ‘harm as a setback to interests’ is a little bit different. Getting hit by a car while riding your bicycle, it’s pretty clear what the harms are, right? Breaking bones, and whatever, those are obvious harms, so that’s how people think of harm. Part of the challenge here is to articulate what the actual harms could be, in a way that’s real. (11, ELSI)

Other interviewees suggested that the possibility of dignitary harm would vary by participant…

For each person, that risk would be very different. Everyone’s going to have some different set of particular uses of their data that they’re going to be concerned about. And some people are not going to be concerned about anything. (31, ELSI)

…including one who pointed to the example of differing reactions to the use of stored materials by commercial companies in particular:

I’m not sure if it’s a risk, it certainly may be a benefit—not a direct benefit, an indirect benefit—for someone, if you have a disease, that there are [companies] working on things. But people sometimes feel very differently about that. Some people don’t care at all and some people get very upset. (50, Human Subjects)

Many thought leaders agreed, however, that the research enterprise itself would ultimately bear substantial adverse consequences if biospecimens and data contributed for research were used in ways objectionable to participants:

When people go into a situation and they have certain expectations and those expectations prove not to be accurate, they lose trust. And they will stop participating. (55, Government)

### Group harm

In general, thought leaders described group harm as a product of research findings that are construed in ways that reflect negatively on particular populations, *“not so much to the individual who’s participating but to the groups of which the participant may be a member”* (22, ELSI):

Even if their individual identity isn’t revealed, sometimes [people] don’t understand that their group membership or their social identity may be important. They may be identified with various groups and research may … make certain statements or claims associated with certain groups. (32, Ethics)

Group harm was often seen as a matter of research results serving to perpetuate or exacerbate existing stereotypes in socially-identifiable populations:

If I were talking to a friend who was part of a particular community that was already more identifiable … or where there were other sensitive issues about certain stereotypes … I would just mention to them that findings that are linked to ancestral information that could be associated with their community have the potential for some who take it out of context to make assumptions about the community as a whole. (21, Government)

Interviewees gave numerous examples of sensitive as well as non-health-related research topics (*e*.*g*., behavioral genetics, intelligence, criminality, substance abuse) that, if differences were found between groups, could easily lend themselves to intensifying stereotypes. As with dignitary harm, they did not perceive group harm as accidental, in that it could result from authorized uses of biospecimens and data, and identified several design features of the Million American Study that could influence the possibility of group harm. For instance, some felt that the massive scale of the study presented no more risk to communities than any other population-based research, or was perhaps even protective:

If it was a study that was *just* studying people from their community, I might encourage them to think about the community risks. But if it’s a study that tries to get people from all different backgrounds and communities, I don’t think that would come up. (16, Ethics)

In contrast, at least one interviewee recommended that vulnerable populations should be studied separately—in conjunction with the larger endeavor, but by researchers with in-depth knowledge of community concerns:

What might actually be more beneficial than the United States government conducting a one million person cohort is to, in addition to that, develop population cohorts of highly-valuable, informative, and sensitive populations—to create cohorts of their own in order to ensure parallel research is happening that will actually benefit those populations… My concern is that the people who will move forward in these things will, in their ignorance, not consider the harms that can occur to a certain population… There’s a thin line between identifying concerns of Native people and stereotyping them—the only way to do this ethically is to engage in the process early on with researchers who have expertise in dealing with the issues that native people are facing. (36, Historically-Disadvantaged)

This interviewee went on to expand on the importance of communities having *“control and at least some power balance and equity*,*”* especially for those *“that have been abused and targeted and have actually a lot to lose”* (36, Historically-Disadvantaged). However, as other thought leaders pointed out, the open-ended design of our hypothetical study provides little participant control over how stored materials are used:

If one is concerned about how [data] could be used to draw conclusions about groups, they should factor that into their decision to participate, as there is no way to prevent it once enrolled. (18, Law)

As with dignitary harm, interviewees considered the integration of oversight bodies and governance processes into the study design to be potentially important…

I would be advising my family members to look very, very carefully at what kinds of oversight mechanisms are present. I’m interested in the oversight mechanisms that protect us from researchers—researchers not doing what they said they were going to do. Protect us from re-identification, but also … make sure that there is robust community input and judgment going on when decisions are made about what kind of research is going to be done. (11, ELSI)

…yet imperfect operational mechanisms to address the risk of group harm:

That’s something that IRBs don’t generally or consistently pay attention to … future social harm, group harms. That’s a very ambiguous area so it would be important for individuals signing up to understand that that is not scrutinized and there’s a potential for that. (32, Ethics)

I have concerns about this outside of personal risk, other risks that are possible … at the level of social harms. I don’t have confidence in the system. I don’t have confidence in the way [the Million American Study] will operate. (49, Research)

With regard to the likelihood and severity of group harm, a few interviewees suggested that these were low:

I could imagine [community-based risks], but I don’t actually think that any of them would rise to the point where I would actually articulate them… People don’t need research to discriminate against all sorts of ethnic groups. So that risk is not one that impresses me. (33, Informatics)

Many thought leaders, however, described group harm as a very real concern. In particular, as one noted, the specter of harm is readily apparent to populations who have experienced it:

People who know nothing about the history of Native people always read or hear the risks as me being hypersensitive, overly sensitive, being suspicious. But with all these things, you have to look at the history and you have to understand where we’re sitting as people who have been colonized and who have been overthrown and are fighting to keep our rights. So unless you have experienced that, you don’t even know how to come up with the risks. (36, Historically-Disadvantaged)

In the context of these discussions about likelihood and severity, a striking number of interviewees, across nearly all categories, referred explicitly to *“the big case—the studies on the material of the Havasupai Native Americans”* (45, Participant-Centric). They also described other real-life examples of notorious research, as well as the racialized translation of research findings (*e*.*g*., BiDil), as the basis for their assessment that group harm was likely:

Certainly we have a very depressing history in biomedical research worldwide and in our country of not being very sensitive to group harm. Look at how African-Americans have fared in Tuskegee … the Guatemala example that was not so long ago … American Indians. I think it’s hard to imagine that you shouldn’t be a little more careful if you’re a member of the community that has traditionally been hurt by research. (44, Research)

Several thought leaders foresaw the consequences of group harm and stigmatization as propagating notions of superior and inferior groups. A few even envisaged eugenics as the most severe outcome:

You’re really making decisions not only on yourself, but also on behalf of your children and your grandchildren and your grandchildren’s grandchildren, and who knows what the society is going to be like in the future and whether another sort of ethnic cleansing kind of approach occurs. We decide that as a society … X-Y-Z is no longer an acceptable trait and we’re going to find those people that have it and … radically discriminate against them. I think it’s a very scary kind of future and I hope we don’t ever encounter that. I think the odds are relatively low that in America we encounter that, but I don’t think it’s zero. (17, Participant-Centric)

Across all of these discussions of group harm, thought leaders observed that racial and ethnic minority groups were more likely than those of European ancestry to experience stigmatization:

If you are a member of an Aboriginal American group, you might well … be very concerned about that risk because that has happened to you and your community many times before. It’s essentially zero likelihood of happening to someone who’s of non-Ashkenazi European background, because that just doesn’t happen anymore. (20, Research)

A few interviewees mentioned groups that might be vulnerable based on other characteristics, such as *“certain disease groups—HIV positive*, *for example*, *people with mental illness*, *people with disability”* (22, ELSI) or socioeconomic status:

I think the harms could be worse in communities of low socioeconomic status. For example, if there are results about a particular … group of people living somewhere, the ones who are not powerful in society, they may be discriminated against more as a result of the group’s results that come out. (10, Historically-Disadvantaged)

Many thought leaders, however, emphasized that all participants might be concerned about their contributions being used for research that stigmatized others:

Because my family is European-American, I think … it’s low risk that my group would be stigmatized. Even so, it would be a very significant risk that my data could still be used in stigmatizing ways… It would certainly be concerning to me and it would be a reason why I might not want to put my data in, even though I would feel personally, or by my group identity, less vulnerable. (11, ELSI)

Not surprisingly, many thought leaders predicted—as they did with dignitary harm—that the research enterprise itself would be detrimentally affected if biospecimens and data were used in ways objectionable to communities: *“I think* [that] *can be really*, *really serious*. *I think it could actually shut down research in certain communities for a really long time”* (30, Historically-Disadvantaged).

### Economic harms

Over half of thought leaders brought up the possibility of economic harms, predominantly from discrimination in employment and health insurance, as well as in other kinds of insurance, that could have direct financial implications.

### Employment and health insurance discrimination

Many thought leaders spoke about the prospect of discrimination by employers and health insurers:

If a person was discovered where they had some condition, unfortunately in our society—not withstanding laws like GINA and non-discrimination acts and things like that—that might lead to actual employment or health insurance type problems. (40, Human Subjects)

For the most part, interviewees did not articulate the route by which research data could get into the hands of employers and health insurers, instead using phrases such as *“loss of privacy”* (42, Ethics), *“loss of confidentiality”* (43, Participant-Centric), *“information that might be revealed”* (51, Informatics), and *“the possibility that somehow your records will get released”* (39, Human Subjects). A few talked about re-identification (*i*.*e*., ascertaining individual identities in data from which identifiers have been removed) as a pathway to discrimination: *“Worst-case scenario*, *somebody with the power to do something bad to you is the one who re-identifies you or who acquires that re-identification data—like your employer or your health insurance company”* (54, Ethics). This interviewee went on to observe, however, that much of the activity around re-identification has been among academic researchers attempting to demonstrate what is possible: *“They’re not interested in trying to publish your name—although in their excitement*, *they can sometimes be careless—they just want to show that they’ve been able to do it*.*”*

A few thought leaders mentioned the possibility of breach as a mechanism by which data could end up being used for discriminatory purposes: *“There is always a chance the computer system will be hacked*, *non-identifiable data will become identifiable*, *there are known examples where this has occurred”* (08, ELSI).

Concerning employment discrimination in particular, one interviewee noted that an avenue by which employers could obtain research data might be if recruitment for our hypothetical study was carried out in the workplace:

Some of my family members that are in the trades are constantly monitored for whether or not they’re doing drugs, and that’s a normal part of the process of getting hired on jobs. While it hasn’t happened that they’ve moved to cigarettes or obesity, there is a feeling at times that they are being discriminated against based on weight and smoking, and certainly other physical disabilities… So I absolutely think if it got into the wrong hands—let’s say that the Million American Study was being done through workplaces, workplaces wanting that information, then using it to maximize their workforce by using that information kind of against people subtly. (05, Research)

Finally, a few thought leaders said that if participants received their individual research results, employers and insurers could gather that information from participants themselves should current laws be changed or weakened:

One of the risks of participating in this study now is that … there are current efforts underway that will lessen people’s protections under GINA and the ADA. So their employer could actually ask them if they or their spouse had such a genetic test. (55, Government)

Regardless of *how* employers and health insurers might gain access to research data, thought leaders typically said the likelihood of harm from this form of discrimination was low. For example: *“Nobody’s going to lose their job or their health insurance or whatever”* (12, Law), *“I give it a low level of likelihood”* (40, Human Subjects), and *“I think it’s pretty unlikely—but not completely impossible*, *so I’d put it pretty low”* (52, ELSI). One compared the prospect of discrimination based on research data to experience with information held in clinical settings, noting that although getting access to the latter *“is much more plausible to do*, *it’s hard to find that there’s been a tsunami of abuse”* (04, Law).

Several interviewees pointed to legal protections currently in place as the reason why the probability of discrimination by employers and health insurers is low:

People are always concerned about employment and insurance discrimination issues. We’ve got GINA and we have the Affordable Care Act, both of which provide some level of protection there. So I think the probabilities of adverse consequences with employment or health insurance are low. (01, Human Subjects)

A few interviewees suggested that, although the likelihood of this kind of discrimination is currently low, it could grow over time as more is learned about the meaning of genetic information:

It’s a very low probability today, but I think it’ll be a growing probability… I do predict there will be industries created just to profile people… I think white collar people don’t realize this, but especially for blue collar jobs and retail industry jobs, there’s a lot of psychometric testing and a lot of profiling by the HR department to see, ‘If I’m going to have to hire 10,000 people, I’m going to try to reduce the risk of badness happening: bad behavior, anti-social behavior, disease, and so on.’ So, I think, as part of HR packages, it is predictable that, in the future, whatever genetic information that is pertinent, they will try to use. (33, Informatics)

Other thought leaders described the likelihood of harm as dependent on the user—what information they have and how they use it (*“It depends on what’s disclosed and how it’s disclosed and who it’s disclosed to”* (25, Historically-Disadvantaged))—or on the circumstances and risk tolerance of the individual who is the subject of the information:

People differ during their lifetimes about their view of privacy, and their risk tolerance toward engaging in research. Studies show that people, once they reach age 65, tend to care less about privacy because they’re not working, they’re not going to lose their job, they’re covered by Medicare, they’re not going to lose access to their health care… The younger folks are often at an immortal stage, and certainly now, when you get to the Millennial generation, they feel more comfortable in having certain kinds of information more widely available than their parents or grandparents would. (12, Law)

Finally, a few interviewees observed that it may be difficult to know or prove that one has, in fact, been harmed by employment or insurance discrimination. For example:

We don’t know what employers are doing… There is EEOC regulation that is supposed to prevent them from discrimination in the workplace based on things like genetic information, but you know, people are savvy enough not to say ‘we’re not going to hire you, or we’re firing you, because of this genetic information about you.’ So that’s a little bit of the unknown and something worth pointing out. This is information about you that could be used in ways that could disadvantage you. We know there are some protections in place but … they’re not perfect and we can’t be sure how imperfect they are. (11, ELSI)

### Other insurance discrimination

Many thought leaders also discussed discrimination in the context of other forms of insurance, including life, disability, and long-term care insurance. They singled out these types of insurance as deserving heightened concern because they are beyond the scope of current protections that apply only to employment and health insurance. Although several interviewees again did not identify the route by which research data could get into the hands of these other types of insurers, many explicitly pointed to return of individual research results to participants as the mechanism, leading to the loss of *“accurate deniability of knowledge of these risks”* (33, Informatics):

The risks you worry about are: finding out something about yourself and from that point forward you now know something about your genetic risk factors you didn’t know before. If somebody asks you about that … there are certain circumstances where you have to make a choice about whether you’re truthful about that. Because before, you didn’t know it and you didn’t have to lie, and then suddenly there may be something… For life insurance, for disability insurance, for long term care insurance—and particularly for long term care because it’s a private voluntary market—the companies are very worried about adverse selection. (52, ELSI)

Incorporating individual research results into medical records was also mentioned as a conduit for insurance companies getting the information: *“If this information is generated and it gets into the individual’s medical record*, *then we can’t rule out that kind of discrimination”* (56, Research).

A few thought leaders mentioned the possibility of breach and re-identification as the means by which life, disability, and long-term care insurers could come into possession of the data:

If somebody was hacking data and giving it to life insurance companies or disability companies … there [is] a danger if this information gets out and it’s used against patients in some way… I still feel like we need to lean towards the side of sharing and learning, rather than not doing that because we might get hacked. Because the truth is, I think we are going to get hacked. (06, Participant-Centric)

Without regard to *how* these kinds of insurers might acquire research data, about half of interviewees who spoke about this kind of discrimination described the likelihood of harm as low (*e*.*g*., *“fairly unlikely”* (43, Participant-Centric), *“a mild chance”* (58, Research)) or theoretical. Some of these pointed to lack of actual occurrences of discrimination, but underscored that there was no particular reason for it not to happen:

We have not seen any negative consequences of that. That doesn’t mean that can’t happen, especially with no protection for genetic information in life insurance and long-term care. It is highly likely that if there’s something that’s negative in that set of information, then you could be denied. (35, Participant-Centric)

One noted that the risk, although currently low, could increase as insurance companies use the results of precision medicine research to refine actuarial tables:

Theoretically you could get discriminated against in some form. We have legislation that prevents certain kinds of discrimination but not all kinds of discrimination. Those odds are relatively low. We know [however] that insurance companies will use the Million Person cohort to do more accurate actuarial tables in a way that is potentially negative to people or to their families. (23, Participant-Centric)

Compared to those who described the potential for harm as low, a roughly equal proportion of thought leaders who spoke about life, disability, and long-term care insurance described the likelihood of discrimination as real and a harm that *“could be serious”* (06, Participant-Centric): *“Certainly on long-term care insurance and life insurance*, *there would be considerable risk”* (59, Government).

One interviewee believed that, although the risk was real, the impact of discrimination in these other types of insurance was less severe than discrimination in employment or health insurance:

I wouldn’t call it catastrophic. Long-term care insurance is basically mitigating the risk of having to pay for your own care at the end of life. I don’t put that on the same level of an entitlement as I do: if you get cancer, you deserve to get treated, or if you have a heart attack, you deserve to get treated. I put it at a slightly lower level of personal need, a little bit lower on Maslow’s hierarchy. So I don’t think it would be catastrophic. But … it is plausible that it may actually evolve into policy. If more and more people have more and more genetic information, I think the insurers are probably going to want to know what you know before they offer you a private voluntary insurance package—and I think it will affect people. It’s a perverse public policy question because the very people who are most likely to develop dementia and need long-term care are the ones that would be shut out of the market. That’s a pretty vexing public policy problem. (52, ELSI)

Others acknowledged a similar point, but went on to describe in further detail how the impact might vary substantially depending on the participant’s health or socioeconomic status:

It depends on the particular participant’s situation. If it’s a healthy person without a chronic disability, something like that, then … it could be serious, but it would probably be less likely to be serious than in the case of somebody who needed long-term care or disability insurance. Life insurance, again, it’s going to be a function of that person’s prior financial situation and what would happen to their family if they died with or without insurance. It’s going to be a very broad distribution—the impact could be negligible or it could be very serious. (43, Participant-Centric)

Other factors thought leaders mentioned as influencing the likelihood of harm included federal and state law being in a state of flux (*“We’re in an era now where the federal law and some state law are actually evolving on this issue*, *so a lot of this will be very contextual”* (59, Government)) and uncertainty regarding cultural reactions to research advances:

On the discrimination side, it’s hard to know what those consequences will be because we haven’t really encountered them as a culture yet, and we don’t really know what the cultural reaction will be. Will the cultural reaction be protective? Will we create rights so that those kinds of discrimination don’t happen? Or will the culture be passive, and allow for that kind of discrimination to be acceptable? (23, Participant-Centric)

### Other economic harms

A few thought leaders suggested other sources of potential economic harm from participation in the Million American Study. For instance, one described discrimination by other entities such as *“mortgage companies*, *or loan companies to deny credit; for example*, *if you are predisposed to developing a disabling condition”* (08, ELSI). Another talked about harms that could have economic implications for particular groups:

The federal government defines who is Alaskan Native or American Indian often by being a member of a federally recognized tribe or by blood quantum. So if some of the … information could be used to determine that maybe somebody was really not Alaskan Native or American Indian, that would have huge ramifications on people’s access to benefits. (30, Historically-Disadvantaged)

Others mentioned the possibility that participants *“could potentially be re-identified in some capacity and … worst case scenario*, *this could lead to such things as identity theft”* (03, Informatics). In general, these interviewees viewed the likelihood of identity theft in a research context as no higher than that in a clinical setting:

The data that we’re getting here is largely contained within your hospitals, by the doctors you see—and they have information systems which are subject really to the same risks as the Million American Study. So you don’t really have a new risk—you just have a new place where that data’s being stored. You already have the risk … and the potential for harm would be mostly around risks of things like identity theft and things being disclosed around who you are. (53, Informatics)

If identity theft were to occur, however, thought leaders gauged the consequences as significantly disruptive—potentially more so than would be caused by unintended access to sensitive health-related information:

There is a market for electronic health records, a black market, but they don’t seem to be worth very much money. I suspect what would be worse and … more disruptive to someone’s life, would be simply the theft of personal data like a Social Security number, that then leads to identity theft, rather than finding out someone has cancer or something. (43, Participant-Centric)

Others portrayed the consequences as serious, although perhaps not disastrous:

[Medical identity theft] would probably be difficult to straighten out. You might have to fight with insurance companies … I think it would be a significant pain. Whether it would be irreparable, I don’t know. It would be very difficult to deal with, probably very similar to financial identity theft. (59, Government)

One interviewee noted that the research enterprise itself would experience the consequences of large-scale re-identification leading to identity theft: *“The seriousness to the project might be that it could be shut down because it would be an example of bad governance or very poor process”* (03, Informatics).

### Psychological harms

About half of thought leaders raised the prospect of psychological harm as a result of return of individual research results, unintended access to stored data, and/or certain design features of the Million American Study. Throughout these discussions, interviewees frequently remarked that psychological implications could extend beyond the participant to other family members:

Your genes don’t tell people just about you. What we learn about you is half information about your siblings, your parents, and your children. You can make the decision about your own information, but you’re also making a decision about information that is relevant to your family members. (14, Law)

### Return of research results

The most common source of psychological harm thought leaders identified was return of individual research results. In particular, several suggested the potential for participants to be distressed by *“finding out things about themselves that they actually didn’t know before and didn’t really want to know”* (27, Government). Others foresaw anxiety based on misunderstanding:

A person might put too much weight on what they see in their genome sequencing, so that if someone sees on the 22nd chromosome or whatever, something that might freak them out even though maybe [the result] triples their [risk] but their chances were one in 6 billion, so now they’re one in 2 billion. The information derived from something like that that may not be fully easily understood by a lay person. (40, Human Subjects)

Some mentioned the psychological sequelae of a false positive result: *“There would probably be a substantial period of anxiety prior to having your true genetic status and risk status clarified”* (01, Human Subjects). Even if a research result is accurate and clinically actionable, on interviewee reflected: *“A person who can’t afford insurance coverage may be more worried about having a genetic test result that they can’t explore further*, *for example*, *with their doctors”* (10, Historically-Disadvantaged).

Several thought leaders noted that the likelihood and severity of psychological harm from receipt of research results would vary based on characteristics of the individual:

It really depends on the person. For some people, the consequences would not be severe at all—distressing perhaps, but people deal with information in very different ways. What might for mildly distressing for one person could be devastating for another person. (27, Government)

This recognition led several interviewees to acknowledge the importance of both eliciting and honoring participant preferences with regard to return of individual results, each of which comes with its own challenges:

The hard thing about constructing these types of research protocols is this is such an individualized thing. The protocol, the research in itself, can have a particular plan of what it wants to do—but the way people want to receive and want this information is just totally different… It’s almost like you need to have people say, ‘I do want to know those things,’ ‘I don’t want to know those things,’ and that’s just not feasible to do. (50, Human Subjects)

The potential to identify risks for genetic disorders that are untreatable and of late onset, where individuals may choose to not want to know that information … I would assume that the study would only return that information upon participant request. But by virtue of being a large study that shares data, there is some risk that that information gets known to the individual despite their request not to know it. (56, Research)

Return of individual research results was also the primary mechanism by which thought leaders anticipated familial distress could occur:

It’s important to remember that information about an individual is not only information about an individual, that it’s information about a family. And you can’t help it if you’re doing something with one individual, if you get certain information it may be very suggestive of a sibling or an offspring or something like that. (50, Human Subjects)

For example, several mentioned results that indicate misattributed parentage as a cause of stress. Family members having different preferences to know or not known certain results was another prominent theme:

There could be family tension if you want to participate and they don’t, or if you … learn something and they don’t want to know, but you feel compelled to tell them, or it’s very difficult to hide. Or some relatives want to know and others don’t. (54, Ethics)

Finally, several thought leaders pointed to the psychological effect of worrying about the implications of one’s own research results for other family members…

I would say one risk is that they will discover things about our family’s genome … that the researchers are unsure about, that doctors might not be able to use to help us, and that that could cause problems. Whether it’s more expensive future testing that we might want done, because now all of a sudden we’re worried about a particular genetic variation, or fear or anxiety and the havoc that could cause to the family if we start finding out that family members have these genes. (10, Historically-Disadvantaged)

…particularly with regard to children and future generations: *“If something is found in you*, *this might cause you to worry about not only chasing it down in yourself but it might cause you to worry about chasing it down in your children”* (16, Ethics).

### Unintended access

As another source of psychological harm, thought leaders discussed the misuse of information gained through unintended access (*e*.*g*., via breach, hacking, or triangulation):

You could imagine someone being outed as having a sexually transmitted disease. You could imagine that getting on to their Facebook page, you could imagine that being used against someone in a dating scenario. Those are pretty bad… I think the word ‘violation’ really fits there. (23, Participant-Centric)

Interviewees acknowledged that the possibility of psychological harm from unintended access relies both on the identifiability of the information…

There is … a risk of other people using your information for bad purposes—if they could get access to information and they knew it was about you. (27, Government)

…as well as a recipient of the information having motivation to use it:

Every once in a while the National Inquirer will have some kind of story about some celebrity, and it was a clerk at the hospital leaked something about someone. (40, Human Subjects)

Several thought leaders further noted that the severity of any psychological harm would depend on the nature of the information, as well as the person.

I would envision that it would probably lead more to embarrassment if there was possibly something in there that they might find embarrassing, but that would be personal. There’s no guarantee that anything would actually be embarrassing. It would be proportional to their expectations for how they keep this information private. (13, Informatics)

### Study design

A few thought leaders referenced the open-ended nature of our hypothetical study and the range of topics that could be studied as a potential source of psychological distress. Some used terms such as *“embarrassment*, *disappointment”* (17, Participant-Centric) to describe the consequences of objectionable research use; more often, interviewees framed this concern as a dignitary harm (see **Dignitary and Group Harms**).

Some thought leaders highlighted the ongoing collection of information from electronic health records as posing *“an overwhelming risk”* (12, Law) of psychological harm—particularly if participants either do not understand or do not remember this design feature of the study:

### Legal harms

Finally, nearly one-fourth of thought leaders mentioned the possibility of legal harm resulting from uses of stored materials that are permitted by the Million American Study, yet beyond the scope of what participants might have expected or would have agreed to at the time of consent. These included uses by the government or law enforcement in ways that could, in some cases, have serious legal implications for participants and even family members. For example, a few thought leaders highlighted the government’s direct involvement in our hypothetical study as a potential source of legal harm:

[If] an academic institution or a private institution held the data, I think the public and my friends and family would probably feel more comfortable that it would not be misused for, for example, surveillance, for law enforcement, or for other types of nefarious purposes that the federal government may have. (42, Ethics)

More commonly, because of the vast size of the Million American Study and the distinguishing nature of genomic information, interviewees observed that the data generated *“could be used even as just a reference for identifying you for something else in the future”* (50, Human Subjects). Specifically, several discussed use of the data by law enforcement:

The primary risks from my point of view are when the information gets into hands that don’t have the same relational ethic or even goal as the original study… One of the biggest fears that I certainly had with [Biobank Name] was that the information would then be used by law enforcement in basic fishing schemes based on DNA found at crime scenes, which actually wreak a huge amount of havoc with people’s lives. (05, Research)

One thought leader warned that *“law enforcement does see it as a repository that they can access… If you build it*, *they will come and they will try to get it”* (55, Government). Another deemed the prospect of law enforcement use as *“pretty darn likely with a million people”* (29, Human Subjects), and went on to note (as did others) that the risk of being ensnared in a criminal investigation extended to family members: *“It’s not just the index individual whose identity is at risk*, [it’s] *the whole biologically-related family whose privacy might be put at risk in that”* (29, Human Subjects). Interviewees reported that these familial consequences had already transpired in the context of other genetic databases:

There’s been information about how some police forces are subpoenaing people for information, genetic information, whether it be from 23andMe or some of these direct-to-consumer sites… Recently, somebody, a dad put his DNA on 23andMe. I don’t know how the police got it, but they got it, and found a familial link and so they went after the son who lived in a different city and he had to give his DNA to prove he wasn’t a suspect. (46, Historically-Disadvantaged)

Regardless of their opinions about the likelihood of legal jeopardy arising from study participation, these thought leaders typically acknowledged that, if such situations do occur, the harm could be *“quite serious”* (29, Human Subjects). Some pointed to the possibility of being falsely implicated…

People are leaving DNA all over the place. You may have been at the bank right before the bank robbers came, but it doesn’t mean that you or your family members were involved. So there’s a whole criminal justice part of DNA that I’m very suspicious of, and some of that is because of the way in which the FBI and other agencies have used DNA kind of inappropriately. (05, Research)

…for which the consequences could be severe: *“If somebody gets convicted of murder … or rape or something like that*, *that’s a really big deal”* (52, ELSI).

## Discussion

Precision medicine research involving the consolidation of multiple types of complex data and widespread sharing for a variety of studies offers the prospect of both benefit and harm. Our study sought to illuminate these by eliciting the perspectives of a diverse group of thought leaders in a range of relevant fields. Interviewees discussed an array of individual and societal benefits, as well as physical, dignitary, group, economic, psychological, and legal harms. Many of these have been discussed in the literature to some degree, although often with a focus on a specific issue (*e*.*g*., identifiability [4, 5]), or from the vantage of a single commentator [14–16] or stakeholder group (*e*.*g*., the general public [17, 18]). Although attention has frequently been focused on genomic information [19–21], concerns raised by genomic data are not unique [22, 23]. Rather, the possibility of certain benefits and harms may be more or less prominent in the context of research involving genomics, or genomic information provides a useful lens for considering issues that are also applicable to other kinds of data.

Descriptive studies such as ours do not tell us what should be done to maximize benefits and minimize potential harms in large-scale precision medicine research. But they do highlight specific challenges that warrant concentrated care during the design, conduct, dissemination, and translation of precision medicine research and in the development of consent materials and processes. We asked thought leaders how they would describe the benefits and harms to family and friends in order to garner the issues they believe people should care about most (rather than, for example, demonstrating for us their knowledge of the literature or understanding of regulatory requirements). We chose to convey our findings through numerous direct quotes so that these prominent individuals could ‘speak for themselves.’ Further, we have presented a combined analysis of all of the benefits and harms raised. Substantial paraphrasing of interviewees’ comments or dividing these findings into separate publications would in some cases have been misleading (*e*.*g*., detaching discussions of harms from benefits). In all cases, it would have led to the loss of informational richness and nuance that is critically needed at this stage in the evolution of precision medicine research. By reporting our interview results in this way, several notable themes emerge across topic areas:

First, returning individual research results was a conspicuous subject throughout. To the extent that participation in precision medicine research offers any prospect of direct health benefit, thought leaders identified return of results as the main avenue by which this could occur. At the same time, return of results was a primary source of nearly every kind of harm. Limiting return to clinically actionable results was seen as a way to guard against the introduction of harm—which would mean that the opportunity for benefit would accrue to only a small fraction of participants and possibly mostly in the future, as more is learned about effective prevention and treatment targeted to specific traits. These findings point to the importance of setting realistic expectations among participants concerning benefit, careful decisions about which results will be offered, and the commensurate allocation of research resources to support an ethical process of return, potentially including individualized interpretation, counseling, and referral [24–28]. It is essential to balance the use of limited research resources for these purposes with two key obligations: maintaining fidelity to the goal of generating generalizable knowledge for the benefit of all, and avoiding the perpetuation of misconceptions about the nature research.

Second, group harm emerged as a striking source of concern. Many thought leaders spontaneously raised issues of dignitary and group harm; others commented in response to our follow-up question about any risks to families and/or communities. We included this prompt in our interview guide for several reasons: group harm has been the subject of relatively less empirical attention, our system of human subjects protections is exclusively focused on individual participants and not long-range effects of applying knowledge gained in research [29], and controversial research has been published that reflects poorly on particular groups [30–33]. According to our interviewees, group harm arising from precision medicine research is a multi-faceted issue that presents the real (as opposed to theoretical) prospect of detriment. Mitigating the problem will require further specification, study, and deliberation, as well as careful attention to complex matters related to the scope of broad consent, research design, and the dissemination and translation of results.

Third, relative to the way risks and harms are currently described in consent forms for precision medicine research [34, 35], the thought leaders we interviewed arguably emphasized a somewhat different set of issues. For example, many seemed to give less weight to the potential for employment and health insurance discrimination and for psychological distress. Further, the probability and/or magnitude of several kinds of harm appeared to be difficult for interviewees to characterize—partly because they are inherently hard to quantify, but also because they are value-laden. Thus, rather than developing consent materials that take a “one-size-fits-all” approach to communicating about benefits and harms, it may be helpful to re-orient at least some aspects of the consent process to assist individuals in evaluating participation decisions in light of their own circumstances and values [36, 37].

Finally, the entity perhaps most susceptible to certain risks is the research enterprise itself [6]. Even in the absence of tangible harm, interviewees expressed concern about serious harm to the public’s willingness to participate in and support biomedical research given occurrences such as a high-profile breach, the use of specimens and data for objectionable purposes, the failure to meet over-sold expectations regarding immediate benefit, or the lack of longer-term commitment to translating research discoveries into improved health, to name just a few. This finding underscores the imperative not only to build and maintain trust but to ensure that individuals, organizations, policies, and processes associated with the research are, in fact, trustworthy [38–40].

Interpretation of our results is subject to several limitations. The approximate proportions of interviewees who discussed different topics should be considered preliminary. These proportions provide an indicator of how commonly various themes arose spontaneously among this diverse group of thought leaders when asked purposely broad, open-ended questions. They do not necessarily provide an accurate forecast of the results if we were to, for example, use our findings to generate a pre-specified list of benefits and harms and ask these individuals or others how much they agree or disagree with each item or to provide closed-ended assessments of the likelihood and magnitude of each benefit and harm.

In addition, essentially none of the prominent individuals we interviewed could be neatly categorized as representing one stakeholder group. Table 1 is an account of the primary perspective for which we identified them as thought leaders, but each could easily have been recognized in two or more categories. For this reason, as well as the qualitative nature of our study, we did not attempt to assess similarities and differences between stakeholder groups. Further investigation of the extent to which perspectives differ between groups, as well as the origins and prevalence of relevant differences may be an area for future research.

We carried out these interviews in 2015–16 in the United States. To a large degree, we believe our results reflect fundamental ethical considerations that endure across time and location. Even so, there have been actual or proposed policy changes in the U.S. in the interim that could alter the real or perceived probability and severity of certain harms [41–45]. Similarly, certain benefits and harms may be gauged differently in other countries, based for example on cultural context and healthcare system differences. More generally, shifting socio-political environments and the swiftly evolving research landscape lead to uncertainties and the prospect of ‘unknown unknowns’ [6]. Ongoing vigilance is needed to ensure that it is safe for people to participate in the long-term endeavors needed to improve health and heath care.

## Supporting information

S1 Appendix Consolidated criteria for reporting qualitative studies (COREQ). (DOCX)

## References

[1] Collins FS, Varmus H. A new initiative on precision medicine. N Engl J Med. 2015;372(9):793–5. 10.1056/NEJMp1500523 25635347 10.1056/NEJMp1500523 PMC5101938

[2] AACR Project GENIE: Powering precision medicine through an international consortium. Cancer Discov. 2017;7(8):818–31. 10.1158/2159-8290.CD-17-0151 28572459 10.1158/2159-8290.CD-17-0151 PMC5611790

[3] National Institutes of Health. All of Us Research Program 2018 [cited 2018 Jul 21]. Available from: https://allofus.nih.gov/.

[4] Rodriguez LL, Brooks LD, Greenberg JH, Green ED. Research ethics. The complexities of genomic identifiability. Science. 2013;339(6117):275–6. 10.1126/science.1234593 23329035 10.1126/science.1234593

[5] Weil CJ, Mechanic LE, Green T, Kinsinger C, Lockhart NC, Nelson SA, et al NCI think tank concerning the identifiability of biospecimens and "omic" data. Genet Med. 2013;15(12):997–1003. 10.1038/gim.2013.40 23579437 10.1038/gim.2013.40 PMC4097316

[6] Beskow LM, Hammack CM, Brelsford KM, McKenna KC. Thought leader perspectives on risks in precision medicine research In: Cohen G, Lynch HF, Vayena E, editors. Big Data, Health Law, and Bioethics. Cambridge, UK: Cambridge University Press; 2018.

[7] Guest G, Bunce A, Johnson L. How many interviews are enough? An experiment with data saturation and variability. Field Methods. 2006;18(1):59–82.

[8] Namey EE, Trotter R. Qualitative research methods In: Guest G, Namey EE, editors. Public health research methods. Los Angeles, CA: Sage Publications; 2015 p. 443–82.

[9] Boeije H. A purposeful approach to the constant comparative method in the analysis of qualitative interviews. Qual Quant. 2002;36(4):391–409.

[10] Corbin JM, Strauss AL. Basics of qualitative research: techniques and procedures for developing grounded theory. 3rd ed Los Angeles, CA: Sage Publications; 2008.

[11] Bernard HR, Ryan GW. Analyzing Qualitative Data: Systematic Approaches. Los Angeles, CA: Sage Publications; 2010.

[12] Guest G, MacQueen KM, Namey EE. Applied Thematic Analysis. Los Angeles, CA: Sage Publications; 2012.

[13] National Bioethics Advisory Commission. Ethical and Policy Issues in Research Involving Human Participants, Volume 1 Rockville, MD: US Government Printing Office; 2001.

[14] Schadt EE. The changing privacy landscape in the era of big data. Mol Syst Biol. 2012;8:612 10.1038/msb.2012.47 22968446 10.1038/msb.2012.47 PMC3472686

[15] Landau S. Control use of data to protect privacy. Science. 2015;347(6221):504–6. 10.1126/science.aaa4961 25635089 10.1126/science.aaa4961

[16] Rothstein MA. Ethical issues in big data health research. J Law Med Ethics. 2015;43(2):425–9. 10.1111/jlme.12258 26242964 10.1111/jlme.12258

[17] Kaufman DJ, Murphy-Bollinger J, Scott J, Hudson KL. Public opinion about the importance of privacy in biobank research. Am J Hum Genet. 2009;85(5):643–54. 10.1016/j.ajhg.2009.10.002 19878915 10.1016/j.ajhg.2009.10.002 PMC2775831

[18] Pullman D, Etchegary H, Gallagher K, Hodgkinson K, Keough M, Morgan D, et al Personal privacy, public benefits, and biobanks: a conjoint analysis of policy priorities and public perceptions. Genet Med. 2012;14(2):229–35. 10.1038/gim.0b013e31822e578f 22261752 10.1038/gim.0b013e31822e578f

[19] Angrist M. Genetic privacy needs a more nuanced approach. Nature. 2013;494(7435):7 10.1038/494007a 23389508 10.1038/494007a

[20] Brenner SE. Be prepared for the big genome leak. Nature. 2013;498(7453):139 10.1038/498139a 23765454 10.1038/498139a

[21] McEwen JE, Boyer JT, Sun KY. Evolving approaches to the ethical management of genomic data. Trends Genet. 2013;29(6):375–82. 10.1016/j.tig.2013.02.001 23453621 10.1016/j.tig.2013.02.001 PMC3665610

[22] Sulmasy DP. Naked bodies, naked genomes: the special (but not exceptional) nature of genomic information. Genet Med. 2015;17(5):331–6. 10.1038/gim.2014.111 25232853 10.1038/gim.2014.111

[23] Rothstein MA. Genetic exceptionalism and legislative pragmatism. J Law Med Ethics. 2007;35(2 Suppl):59–65. 10.1111/j.1748-720X.2007.00154.x 17543059 10.1111/j.1748-720X.2007.00154.x

[24] Fabsitz RR, McGuire A, Sharp RR, Puggal M, Beskow LM, Biesecker LG, et al Ethical and practical guidelines for reporting genetic research results to study participants: updated guidelines from a National Heart, Lung, and Blood Institute working group. Circ Cardiovasc Genet. 2010;3(6):574–80. 10.1161/CIRCGENETICS.110.958827 21156933 10.1161/CIRCGENETICS.110.958827 PMC3090664

[25] Wolf SM, Crock BN, Van Ness B, Lawrenz F, Kahn JP, Beskow LM, et al Managing incidental findings and research results in genomic research involving biobanks and archived data sets. Genet Med. 2012;14(4):361–84. 10.1038/gim.2012.23 22436882 10.1038/gim.2012.23 PMC3597341

[26] Knoppers BM, Deschenes M, Zawati MH, Tasse AM. Population studies: return of research results and incidental findings Policy Statement. Eur J Hum Genet. 2013;21(3):245–7. 10.1038/ejhg.2012.152 22781095 10.1038/ejhg.2012.152 PMC3573193

[27] Jarvik GP, Amendola LM, Berg JS, Brothers K, Clayton EW, Chung W, et al Return of genomic results to research participants: the floor, the ceiling, and the choices in between. Am J Hum Genet. 2014;94(6):818–26. 10.1016/j.ajhg.2014.04.009 24814192 10.1016/j.ajhg.2014.04.009 PMC4121476

[28] Budin-Ljosne I, Mascalzoni D, Soini S, Machado H, Kaye J, Bentzen HB, et al Feedback of individual genetic results to research participants: is it feasible in Europe? Biopreserv Biobank. 2016;14(3):241–8. 10.1089/bio.2015.0115 27082461 10.1089/bio.2015.0115 PMC4913503

[29] U.S. Department of Health and Human Services. Protection of Human Subjects (45 CFR 46) 2009.

[30] Hayden EC. Ethics: Taboo genetics. Nature. 2013;502(7469):26–8. 10.1038/502026a 24091964 10.1038/502026a

[31] Piffer D. A review of intelligence GWAS hits: Their relationship to country IQ and the issue of spatial autocorrelation. Intelligence. 2015;53:43–50.

[32] Långström N, Babchishin KM, Fazel S, Lichtenstein P, Frisell T. Sexual offending runs in families: A 37-year nationwide study. Int J Epidemiol. 2015;44(2):713–20. 10.1093/ije/dyv029 25855722 10.1093/ije/dyv029 PMC4469797

[33] Tiihonen J, Rautiainen MR, Ollila HM, Repo-Tiihonen E, Virkkunen M, Palotie A, et al Genetic background of extreme violent behavior. Mol Psychiatry. 2015;20(6):786–92. 10.1038/mp.2014.130 25349169 10.1038/mp.2014.130 PMC4776744

[34] Khan A, Capps BJ, Sum MY, Kuswanto CN, Sim K. Informed consent for human genetic and genomic studies: a systematic review. Clin Genet. 2014;86(3):199–206. 10.1111/cge.12384 24646408 10.1111/cge.12384

[35] Henderson GE, Wolf SM, Kuczynski KJ, Joffe S, Sharp RR, Parsons DW, et al The challenge of informed consent and return of results in translational genomics: empirical analysis and recommendations. J Law Med Ethics. 2014;42(3):344–55. 10.1111/jlme.12151 25264092 10.1111/jlme.12151 PMC4262925

[36] Brehaut JC, Lott A, Fergusson DA, Shojania KG, Kimmelman J, Saginur R. Can patient decision aids help people make good decisions about participating in clinical trials? A study protocol. Implement Sci. 2008;3:38 10.1186/1748-5908-3-38 18651981 10.1186/1748-5908-3-38 PMC2517068

[37] Brehaut JC, Fergusson DA, Kimmelman J, Shojania KG, Saginur R, Elwyn G. Using decision aids may improve informed consent for research. Contemp Clin Trials. 2010;31(3):218–20. 10.1016/j.cct.2010.02.002 20156597 10.1016/j.cct.2010.02.002

[38] Meslin EM, Cho MK. Research ethics in the era of personalized medicine: updating science's contract with society. Public Health Genomics. 2010;13(6):378–84. 10.1159/000319473 20805701 10.1159/000319473 PMC2951727

[39] O'Doherty KC, Burgess MM, Edwards K, Gallagher RP, Hawkins AK, Kaye J, et al From consent to institutions: Designing adaptive governance for genomic biobanks. Soc Sci Med. 2011;73(3):367–74. 10.1016/j.socscimed.2011.05.046 21726926 10.1016/j.socscimed.2011.05.046

[40] Burke W, Beskow LM, Trinidad SB, Fullerton SM, Brelsford KM. Informed consent in translational genomics: insufficient without trustworthy governance. J Law Med Ethics. 2018;46(1):79–86. 10.1177/1073110518766023 29962827 10.1177/1073110518766023 PMC6023399

[41] Rothstein MA. The end of the HIPAA Privacy Rule? J Law Med Ethics. 2016;44(2):352–8. 10.1177/1073110516654128 27338610 10.1177/1073110516654128

[42] Oliphant EN, Terry SF. GINA and ADA: New rule seriously dents previous protections. Genet Test Mol Biomarkers. 2016;20(7):339–40. 10.1089/gtmb.2016.29017.sjt 27336709 10.1089/gtmb.2016.29017.sjt

[43] Hudson KL, Collins FS. The 21st Century Cures Act—a view from the NIH. N Engl J Med. 2017;376(2):111–3. 10.1056/NEJMp1615745 27959585 10.1056/NEJMp1615745 PMC6688288

[44] McClellan M, Japinga M. The Affordable Care Act: What's next? Annu Rev Med. 2018;69:41–52. 10.1146/annurev-med-061516-112359 29414261 10.1146/annurev-med-061516-112359

[45] Underhill K. Raising the stakes for nondiscrimination protections in the ACA. Hastings Cent Rep. 2018;48(1):8–9. 10.1002/hast.805 29457243 10.1002/hast.805

