# Supplementary material for: Thought leader perspectives on benefits and harms in precision medicine research
Source: PLoS One. 2018 Nov 26;13(11):e0207842. doi: 10.1371/journal.pone.0207842 (PMC6258115; doi:10.1371/journal.pone.0207842)
Supplement: S1 Appendix — (DOCX) [file pone.0207842.s001.docx]

**S1 Appendix. Consolidated criteria for reporting qualitative studies (COREQ)**

| **Domain 1: Research Team and Reflexivity** |  |
| --- | --- |
| **PERSONAL CHARACTERISTICS** |  |
| 1. Interviewer/facilitator: Which author/s conducted the interview or focus group? | The interviews were conducted under the leadership of the Principal Investigator, Laura Beskow (author) by Catherine Hammack (author), Kathleen Brelsford (author), and Kevin McKenna (acknowledged). |
| 2. Credentials: What were the researcher’s credentials? (*e.g. PhD, MD)* | Laura Beskow, MPH, PhD; Professor; female; health policy, research ethics  Catherine Hammack, JD, MA; Social Scientist; female; law, bioethics  Kathleen Brelsford, PhD, MPH; Senior Social Scientist; female; medical anthropology  (Kevin McKenna, MPH; Qualitative Analyst; male; public health) |
| 3. Occupation: What was their occupation at the time of the study? |  |
| 4. Gender: Was the researcher male or female? |  |
| 5. Experience and training: What experience or training did the researcher have? | Each team member has at least ten years of research experience and extensive training in qualitative techniques (including the conduct of semi-structured interviews and qualitative coding and analysis). |
| **RELATIONSHIP WITH PARTICIPANTS** |  |
| 6. Relationship established: Was a relationship established prior to study commencement? | No relationship was established between an interviewee and interviewer prior to study commencement. |
| 7. Participant knowledge of the interviewer: What did the participants know about the researcher? *(e.g., personal goals, reasons for doing the research)* | Prospective participants were provided with information about funding source, the overall goals of the study, and the specific goals of the interviews. |
| 8. Interviewer characteristics: What characteristics were reported about the interviewer/facilitator? *(e.g., bias, assumptions, reasons and interests in the research topic)* | No interviewer characteristics were reported to interviewees. |
| **Domain 2: Study Design** |  |
| **THEORETICAL FRAMEWORK** |  |
| 9. Methodological orientation and Theory: What methodological orientation was stated to underpin the study? *(e.g., grounded theory, discourse analysis, ethnography, phenomenology, content analysis)* | We used an over-arching grounded theory research methodology. Within the overall framework, we employed an applied thematic analysis (including constant comparative analysis) to identify and refine meaningful categories. |
| **PARTICIPANT SELECTION** |  |
| 10. Sampling: How were participants selected? *(e.g., purposive, convenience, consecutive, snowball)* | Purposive and referral sampling, as described under Methods-Participants |
| 11. Method of approach: How were participants approached? *(e.g. face-to-face, telephone, mail, email)* | Prospective participants were approached by email. |
| 12. Sample size: How many participants were in the study? | n = 60 |
| 13. Non-participation: How many people refused to participate or dropped out? Reasons? | Among the 95 eligible individuals invited, 35 did not complete an interview. Among these:   - 3 declined - 3 said ‘yes’ but did not respond to our attempts to schedule the interview - 5 said they were unavailable (e.g., too busy*) - 24 did not respond - No one dropped out   (*One person who was unavailable because on family leave later contacted us to volunteer for an interview, but we had completed data collection by that time.) |
| **SETTING** |  |
| 14. Setting of data collection: Where was the data collected? *(*e*.g., home, clinic, workplace)* | Interviews were conducted by telephone. |
| 15. Presence of non-participants: Was anyone else present besides the participants and researchers? | No |
| 16. Description of sample: What are the important characteristics of the sample? *(e.g., demographic data, date)* | The sample is described in detail under Methods-Participants and under Results-Participant Characteristics (Table 1). |
| **DATA COLLECTION** |  |
| 17. Interview guide: Were questions, prompts, guides provided by the authors? Was it pilot tested? | The interview questions and prompts associated with the data reported here are provided (Methods-Instrument Development); the entire interview guide is available upon request. The interview guide was pilot tested. |
| 18. Repeat interviews: Were repeat interviews carried out? If yes, how many? | No interviews were repeated. |
| 19. Audio/visual recording: Did the research use audio or visual recording to collect the data? | With participants’ permission, interviews were digitally recorded. Three participants declined to be recorded but did agree that detailed notes could being taken during the interview. |
| 20. Field notes: Were field notes made during and/or after the interview or focus group? | Yes |
| 21. Duration: What was the duration of the interviews or focus group? | On average, each interview lasted ~1 hour. |
| 22. Data saturation: Was data saturation discussed? | Coding was conducted iteratively. Additional codes were added to the codebook in cases where new ideas emerged. After completing 52 interviews, no additional themes were identified to add to the codebook, suggesting saturation. However, given that we had identified 9 groups of TLs, and expected a minimum of 6 interviewees in each category to achieve saturation, we continued sampling to 60 (the point at which we had interviewed at least 6 individuals per group). |
| 23. Transcripts returned: Were transcripts returned to participants for comment and/or correction? | No |
| **Domain 3: Analysis and Findings** |  |
| **DATA ANALYSIS** |  |
| 24. Number of data coders: How many data coders coded the data? | Two |
| 25. Description of the coding tree: Did authors provide a description of the coding tree? | The systematic application of structural and content codes is described under Methods-Data Analysis. The headings and subheadings used in the manuscript reflect the basic structure of the coding tree. |
| 26. Derivation of themes: Were themes identified in advance or derived from the data? | Themes were derived from the data. |
| 27. Software: What software, if applicable, was used to manage the data? | NVivo 11 |
| 28. Participant checking: Did participants provide feedback on the findings? | No |
| **REPORTING** |  |
| 29. Quotations presented: Were participant quotations presented to illustrate the themes / findings? Was each quotation identified? *(e.g., participant number)* | Participant quotations were presented and each quote was identified by participant number. |
| 30. Data and findings consistent: Was there consistency between the data presented and the findings? | Our manuscript integrates extensive use of direct quotes to provide evidence for each conclusion drawn. |
| 31. Clarity of major themes: Were major themes clearly presented in the findings? | Major themes are clearly identified by headings and subheadings. |
| 32. Clarity of minor themes: Is there a description of diverse cases or discussion of minor themes? | There is substantial discussion of themes within each subheading, including diverse cases and minority opinions. |
